# Fungal Saprotrophic Promotion and Plant Pathogenic Suppression under Ditch-Buried Straw Return with Appropriate Burial Amount and Depth

**DOI:** 10.3390/plants13131738

**Published:** 2024-06-24

**Authors:** Jie Zhou, Yanling Li, Jiawen Lou, Yuekai Wang, Zhengrong Kan, Reinhard W. Neugschwandtner, Fengmin Li, Jian Liu, Ke Dong, Yaguang Xue, Haishui Yang, Lingling Shi

**Affiliations:** 1 College of Agriculture, Nanjing Agricultural University, Nanjing 210095, China; jiezhou516@hotmail.com (J.Z.); liyanling@njau.edu.cn (Y.L.); 18305177769@163.com (J.L.); ykwang@njau.edu.cn (Y.W.); kzr@njau.edu.cn (Z.K.); fmli@njau.edu.cn (F.L.); 2 Longkang Farm, Anhui Agricultural Reclamation Group Co., Ltd., Huaiyuan 233426, China; 3 Institute of Agronomy, University of Natural Resources and Life Sciences Vienna (BOKU), 3430 Tulln, Austria; reinhard.neugschwandtner@boku.ac.at; 4 Institute of Agricultural Sciences of Yanjiang District of Jiangsu Province, Rugao 226541, China; ntliuj@sina.com; 5 Life Science Major, Kyonggi University, Suwon 16227, Republic of Korea; dongke@kgu.ac.kr; 6 Jiangsu Key Laboratory for Information Agriculture, Nanjing Agricultural University, Nanjing 210095, China; 7 Jiangsu Collaborative Innovation Center for Modern Crop Production, Nanjing Agricultural University, Nanjing 210095, China; 8 Geo-Biosphere Interactions, Department of Geosciences, Faculty of Sciences, University of Tuebingen, 72074 Tuebingen, Germany; shilingling@mail.kib.ac.cn

**Keywords:** rice–wheat cropping, straw return, deep tillage, soil fungal community, enzyme activity

## Abstract

Fungi as heterotrophs are key participants in the decomposition of organic materials and the transformation of nutrients in agroecosystems. Ditch-buried straw return as a novel conservation management strategy can improve soil fertility and alter hydrothermal processes. However, how ditch-buried straw return strategies affect the soil fungal community is still unclear. Herein, a 7-year field trial was conducted to test the influences of burial depth (0, 10, 20, 30, and 40 cm) and the amount of ditch-buried straw (half, full, double) on the diversity, composition, and predicted functions of a soil fungal community, as well as the activities of carbon-degraded enzymes. Under the full amount of straw burial, the abundance of phylum *Ascomycota* was 7.5% higher as compared to other burial amount treatments. This further increased the activity of cellobiohydrolase by 32%, as revealed by the positive correlation between *Ascomycota* and cellobiohydrolase. With deeper straw burial, however, the abundance of *Ascomycota* and β-D-glucopyranoside activity decreased. Moreover, genus *Alternaria* and *Fusarium* increased while *Mortierella* decreased with straw burial amount and depth. *FUNgild* prediction showed that plant fungal pathogens were 1- to 2-fold higher, whilst arbuscular mycorrhizal fungi were 64% lower under straw buried with double the amount and at a depth of 40 cm. Collectively, these findings suggest that ditch-buried straw return with a full amount and buried at a depth less than 30 cm could improve soil nutrient cycles and health and may be beneficial to subsequent crop production.

## 1. Introduction

Straw return is an important practice for increasing soil organic matter in agroecosystems [1,2]. This can improve soil structure and diversify nutrient supply [3,4]. Soil microorganisms play important roles in the degradation of straw through releasing cellulase and ligninase [5,6]. Simultaneously, the returned straw supplies plentiful carbon and energy for soil microorganisms, thus promoting their growth and activity [7,8,9]. Since soil microbial community structure has been widely used as an indicator of soil quality [10,11], it deserves more efforts to decipher how straw incorporation influences soil microbial communities and functioning.

As one of the largest contributors to global biomass, soil-inhabiting fungi perform crucial roles in sustaining soil health, nutrient cycling, and crop productivity in terrestrial ecosystems [12,13]. For instance, symbiotic fungi could stimulate nutrient transfer, e.g., nitrogen and phosphorus to crops from the soils beyond the rhizosphere [14,15]. Fungal decomposers could recycle soil organic matter and mineral nutrients [16]. However, pathogenic fungi would passively influence plant growth and subsequently productivity [17]. Given that soil fungi are sensitive to anthropogenic disturbances such as tillage and straw incorporation [18,19], variations in soil fungal community structure may therefore influence their functioning, such as soil organic matter decomposition and nutrient utilization, as well as the acquisition by crops, which can directly affect soil health and crop productivity [20,21].

Soil fungi can excrete a comprehensive set of enzymes to degrade organic materials and thus play a major role in the recycling and reutilization of straw residues [22,23]. Yet, there are few studies focusing on fungal ecology and functioning in response to straw return practices. For example, Huang et al. [24] reported that soil fungal diversity was increased by straw returning, and a larger enhancement was observed at a higher rate of returned straw. Soil fungal community changed after rice straw incorporation, particularly for cellulose-decomposing fungi, which became dominant [25,26]. Although straw carries plant-parasitic fungi [27], ditch-buried straw return increased the abundance of symbiotic fungi, whilst decreasing the pathotrophic fungal abundance [28]. Despite these findings, it is not well known how fungal community composition and functional guilds change with different straw return strategies.

Rice–wheat rotation is the major farming system in Eastern China, with an annual wheat and rice yield of ~4500 kg ha^−1^ and ~9000 kg ha^−1^, respectively [29]. The effective management of straw residues is therefore quite important for the development of sustainable agriculture. Ditch-buried straw return (DB-SR) is a novel conservation management strategy that can combine deep ploughing (10% ditching) and no till (90% without ditching) into one rotational tillage regime in the same cropping season [30]. Our previous findings demonstrated that DB-SR can enhance soil carbon accumulation and nutrient availability, alter hydrothermal processes, and improve crop growth and yield performance [30]. However, how different DB-SR strategies, e.g., burial amount and depth, affect soil fungal community structure and functioning is still unclear. We therefore conducted two 7-year field experiments with different burial amounts and depths in a rice–wheat double cropping system under DB-SR practice in the eastern China. We hypothesized that (1) with a greater amount of straw returned to the soils, fungal community structure would be altered and then stimulate the production of C-degraded enzymes; (2) with deeper buried straw, more pathogenic fungi would be stimulated. We analyzed the diversity, composition, functional guilds, and C-degrading enzymatic activity of soil fungal communities.

## 2. Materials and Methods

### 2.1. Site Description

A field experiment was performed at the Nantong Academy of Agricultural Sciences, Jiangsu Province (32°13′ N, 120°63′ E). The region belongs to a subtropical monsoonal climate, with an average annual temperature and precipitation of 14.4 °C and 1057 mm, respectively. Before starting the experiment, soil properties were as follows: bulk density = 1.4 g cm^−3^, pH = 6.7, soil organic C (SOC) = 11.86 g kg^−1^, total nitrogen (TN) = 1.62 g kg^−1^, available phosphorus (AP) = 12.76 mg kg^−1^.

### 2.2. Experimental Design

#### 2.2.1. Experiment 1: Straw Burial Depth

A field experiment was started in November 2008 with a random block design, including five treatments: straw removal (Control) and ditch-buried straw return to depths of 10 cm, 20 cm, 30 cm, and 40 cm. Each treatment had 3 replications, yielding 15 plots in total with a size of 18 m^2^ (3 × 6 m). For ditch-buried treatments, straw ditches were first manually created to corresponding depths (10 cm, 20 cm, 30 cm, and 40 cm) with a width of 20 cm and interval of 2 m between adjacent ditches. The intact straws were then manually returned to the bottom of the ditches and covered with soil from ditching. The amount of straw returned to rice and wheat was 9000 kg ha^−1^ and 4500 kg ha^−1^, respectively.

#### 2.2.2. Experiment 2: Straw Burial Amount

A field experiment was started in November 2008 with random block design which contained three treatments: straw removal (Control) and DB-SR with half straw amount (H), full straw amount (F), and double straw amount (D). Each treatment was replicated 3 times, yielding 12 plots in total with a size of 18 m^2^ (3 × 6 m). All treatments were conducted at a burial depth of 20 cm. Half straw amounts were 4500 kg ha^−1^ for rice and 2250 kg ha^−1^ for wheat. Full straw amounts were 9000 and 4500 kg ha^−1^ for rice and wheat, respectively, which represented the whole amount of straw residues produced at the current farming level. Double straw amounts were 18,000 kg ha^−1^ for rice and 9000 kg ha^−1^ for wheat, which were designed for the future high or superhigh yielding level. The agronomic managements were similar between the two field experiments except for straw practices. The rice variety was Nangeng 5055, and the wheat variety was Yangmai 13. The applied amounts of N fertilizer were 300 and 180 kg ha^−1^ for rice and wheat seasons, respectively. The P fertilizer (P_2_O_5_) was applied at rates of 180 and 80 kg ha^−1^ for rice and wheat seasons, and the K fertilizer (K_2_O) was applied at rates of 250 and 150 kg ha^−1^ for rice and wheat seasons, respectively.

### 2.3. Soil Sample and Measurements

Soil was collected from top 10 cm depth in May 2015. The location of the straw ditch was first labeled. Soil cores were sampled at the middle position of each straw ditch, and then mixed thoroughly as one composite sample. Soils were passed through a 2 mm sieve to remove undecomposed straws, root debris, and small stones and then separated into three parts. One sub-sample was stored at −20 °C for the determination of DNA sequencing; the second part was stored at 4 °C for NO_3_^−^ and NH_4_^+^ measurement; and the other sub-sample was air-dried to determine pH, AP, and AK. The contents of soil NO_3_^−^ and NH_4_^+^ were analyzed using a San++ Continuous Flow Analyzer (Skalar, Breda, The Netherlands). The concentration of soil AP was determined by the molybdenum blue colorimetric assay, and the concentration of AK was determined by flame photometry.

### 2.4. Enzyme Activity Analysis

The activities of β-D-Glucosidase, cellobiohydrolase, and peroxidase were determined according to the methods described by Tiemann and Grandy [31]. Briefly, one gram of fresh soil was mixed with buffer (pH = 6.5), and labile substrates were added to the buffer to incubate at 25 °C for 18 h before analyzing fluorescence for β-D-Glucosidase and cellobiohydrolase, as well as determining absorbance for peroxidase on a Synergy HT plate reader (BioTek, Winooski, VT, USA).

### 2.5. DNA Extraction

The DNA was extracted from 0.5 g of each soil using a Fast DNA SPIN Kit for Soil (Illumina, San Diego, CA, USA). DNA quality was tested by 1.5% agarose gel electrophoresis in 1× TAE buffer, and the concentration of DNA was determined using an ND-1000 spectrophotometer (Nanodrop Technology, Wilmington, NC, USA). The extracted DNA was stored at −80 °C before PCR amplification. The fungal ITS rDNA gene was amplified with the primer pair ITS1F (5′-barcode-CTTGGTCATTTAGAGG AAGTAA-3′) and 2043R (5′-GCTGCGTTCT TCATCGATGC-3′) [32]. For detailed determination, please see the Supplementary Information. Operational taxonomic units (OTUs) were clustered with 97% similarity cutoff using UPARSE (version 7.1, http://drive5.com/uparse/ (accessed on 20 January 2024)), and chimeric sequences were identified and removed using UCHIME v.4.2.40 [33].

### 2.6. Statistical Analysis

After confirming the normal distribution and homogeneity of variances, the analysis of variance (ANOVA) was performed to compare the means of variables among treatments using SPSS v.19.1 software (SPSS Inc., Chicago, IL, USA). Principal coordinate analysis (PCoA) was used to illustrate the dissimilarities among fungal communities using Bray–Curtis distance constructed on the OTU table, which was performed using the VEGAN package in R (version 4.1.3). To evaluate the relationships between fungal community structure and soil physicochemical properties, redundancy analysis (RDA) was conducted in Canoco v5.0. The ecological function categories of fungi were predicted with the FUNGuild database (https://github.com/UMNFuN/FUNGuild (accessed on 20 January 2024)) based on relative abundance at the OTU level. This step was performed on the FUNGuild website. According to the results of the confidence assessment of the FUNGuild database, only the confidence levels “highly probable” and “probable” were used for subsequent analysis.

## 3. Results

### 3.1. Soil Fungal Community Diversity and Composition

**Figure 1:**
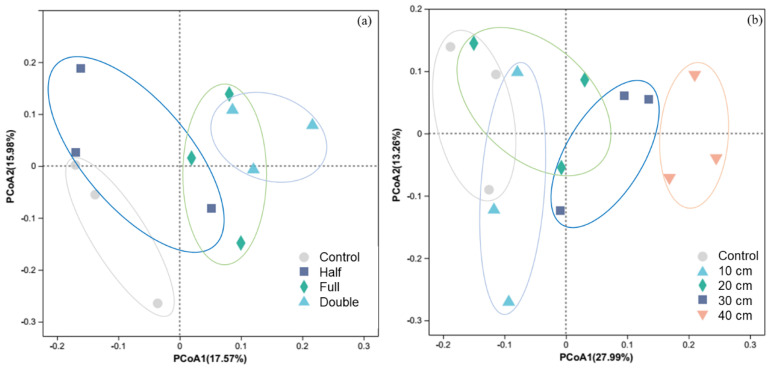
Principal coordinate analysis (PCoA) of soil fungi community structure in response to straw return amount (**a**) and buried depth (**b**). Control, no tillage and no straw return; 10 cm, straw return with full amount return at a depth of 10 cm; 20 cm, straw return with full amount return at a depth of 20 cm; 30 cm, straw return with full amount return at a depth of 30 cm; 40 cm, straw return with full amount return at a depth of 40 cm; Half, straw return with half amount; Full, straw return with full amount; Double, straw return with double amount.

Regardless of DB-SR amount or depth, soil fungal diversity was not affected (*p* > 0.05, Figure S1). However, PCoA results showed that soil fungal community composition under DB-SR with variable amounts differed from control (*p* < 0.05, Figure 1a). Similarly, straw burial depth at 30 and 40 cm altered soil fungal community composition in relative to burial depth at 10 and 20 cm (*p* < 0.05, Figure 1b).

**Figure 2:**
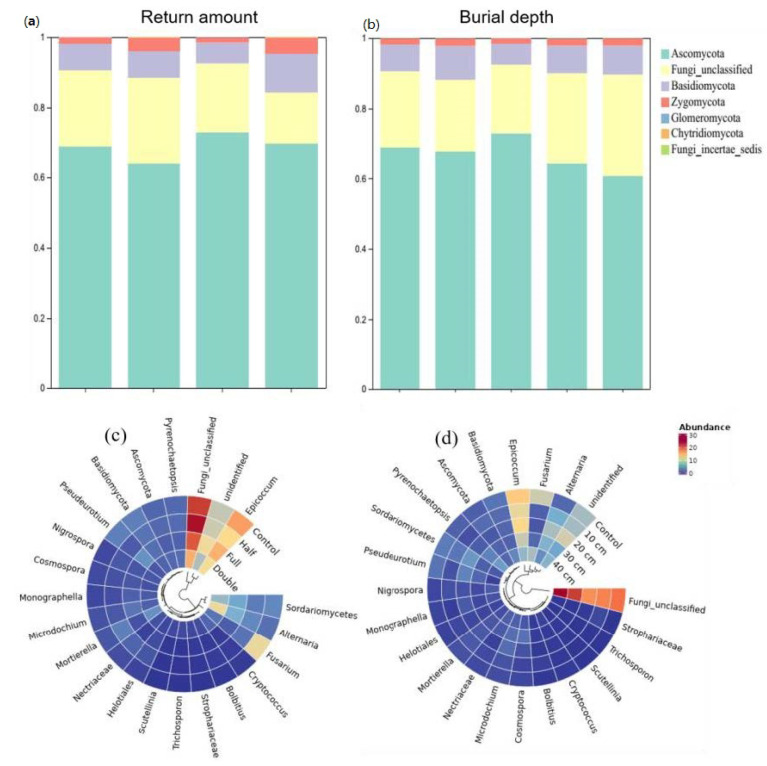
Relative abundances of soil fungi community composition under different straw returned amounts and burial depth managements at the phylum (**a**,**c**) and genus levels (**b**,**d**). Control, no tillage and no straw return; 10 cm, straw return with full amount return at a depth of 10 cm; 20 cm, straw return with full amount return at a depth of 20 cm; 30 cm, straw return with full amount return at a depth of 30 cm; 40 cm, straw return with full amount return at a depth of 40 cm; Half, straw return with half amount; Full, straw return with full amount; Double, straw return with double amount.

**Figure 3:**
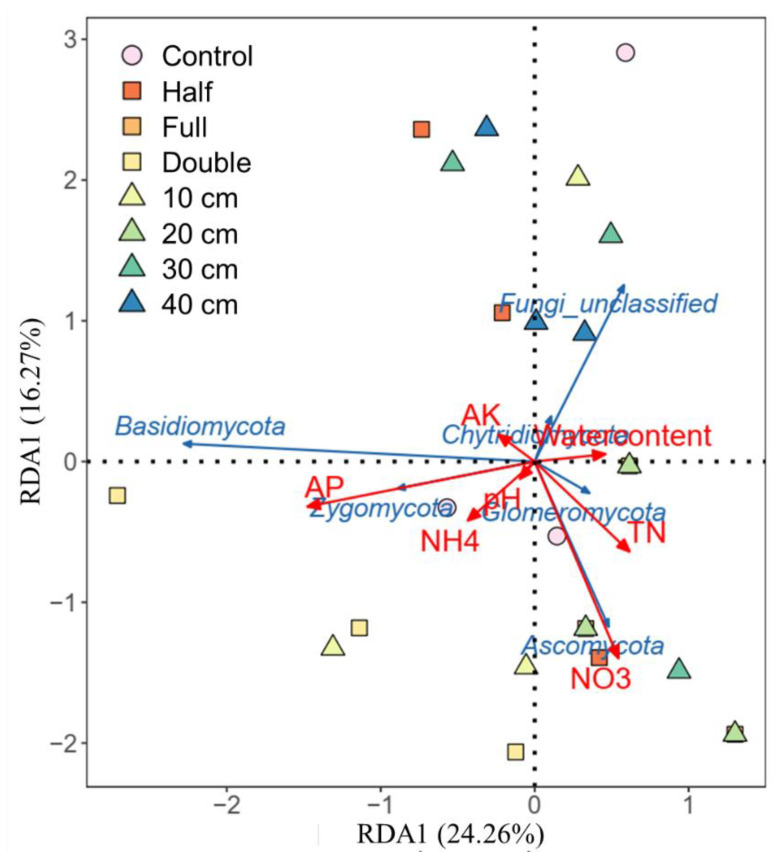
Effects of soil chemical properties on soil fungi community composition based on distance-based redundancy analysis (RDA) in response to straw management practices. TN, total N; NH_4_, Ammonia N; NO_3_, nitrate N; AP, available phosphorus; AK, available potassium.

Compared to control, straw buried at 20 cm increased the relative abundance of *Ascomycota* by 8.2% and *Basidiomycota* by 62% (*p* < 0.05, Figure 2a). The relative abundance of *Zygomycota* was 25%, 16%, and 18% higher under straw buried at 10, 30, and 40 cm than that under control, respectively. Also, straw buried at 20 cm decreased the abundance of *Basidiomycota* and *Zygomycota* but increased the abundance of *Ascomycota* relative to other straw burial depth treatments. In addition, straw buried at 10, 20, 30, and 40 cm increased the abundance of genus *Microdochium* and *Nigrospora*, while it decreased the relative abundance of *Epicoccum*, *Fusarium,* and *Humicola* (Figure 2c). Relative to control, straw with a half amount increased the abundance of *Ascomycota* by 7.5% but decreased *Zygomycota* by 133% (*p* < 0.05, Figure 2b). On the contrary, straw with double amount increased the abundance of *Basidiomycota* but decreased *Zygomycota*. Compared to half and double straw return treatments, DB-SR-F increased the abundance of *Ascomycota* while it decreased *Basidiomycota* and *Chytridiomycota* (*p* < 0.05). Moreover, NO_3_- and TN were positively correlated with *Ascomycota* and *Glomeromycota*, and AP was positively correlated to *Basidiomycota* and *Zygomycota* (*p* < 0.05, Figure 3).

### 3.2. Soil Fungal Function

**Figure 4:**
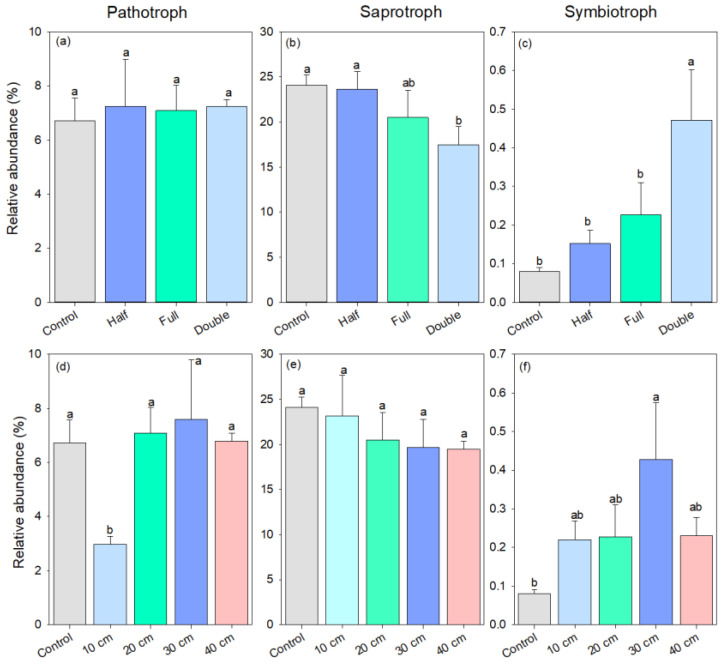
Relative abundance of soil fungal functional groups under different straw management practices at trophic mode level under FUNGuild: (**a**,**d**) pathotroph; (**b**,**e**) saprotroph; (**c**,**f**) symbiotroph. Control, no tillage and no straw return; 10 cm, straw return with full amount return at a depth of 10 cm; 20 cm, straw return with full amount return at a depth of 20 cm; 30 cm, straw return with full amount return at a depth of 30 cm; 40 cm, straw return with full amount return at a depth of 40 cm; Half, straw return with half amount; Full, straw return with full amount; Double, straw return with double amount. Different letters indicate significant differences between treatments (*p* < 0.05).

As for fungal trophic mode, the relative abundance of pathotrophic fungi was similar among half and full DB-SR amount treatments and control (*p* > 0.05, Figure 4a). However, DB-SR with a double amount increased the abundance of plant fungal pathogens by 2-fold and decreased the abundance of fungal saprotrophs by 27% relative to DB-SR with full amount return (*p* < 0.05, Figure 4b). The abundance of symbiotrophic fungi was 90–489% higher under DB-SR amount treatments than control, but it was 52% lower under DB-SR with full amount relative to double amount (*p* < 0.05, Figure 4c). Regarding DB-SR depth treatments, The abundance of fungal saprotroph was 15–22% lower under straw buried at 20, 30 cm, and 40 cm than under control (*p* < 0.05, Figure 4e). By contrast, the abundance of symbiotrophic fungi was 1.7 to 4.3 times higher under DB-SR depth treatments as compared to control (Figure 4f).

As for fungal guild, DB-SR with double amount increased the abundance of plant fungal pathogens by 2-fold and decreased the abundance of arbuscular mycorrhizal fungi by 45% relative to DB-SR with full amount return (*p* < 0.05, Figure S3a,b). Additionally, straw buried at 40 cm increased the abundance of plant fungal pathogens by 52–97% whilst it decreased the abundance of arbuscular mycorrhizal by 64% compared with straw buried at 30 cm (Figure S3c).

### 3.3. C-Degrading Enzymes

**Figure 5:**
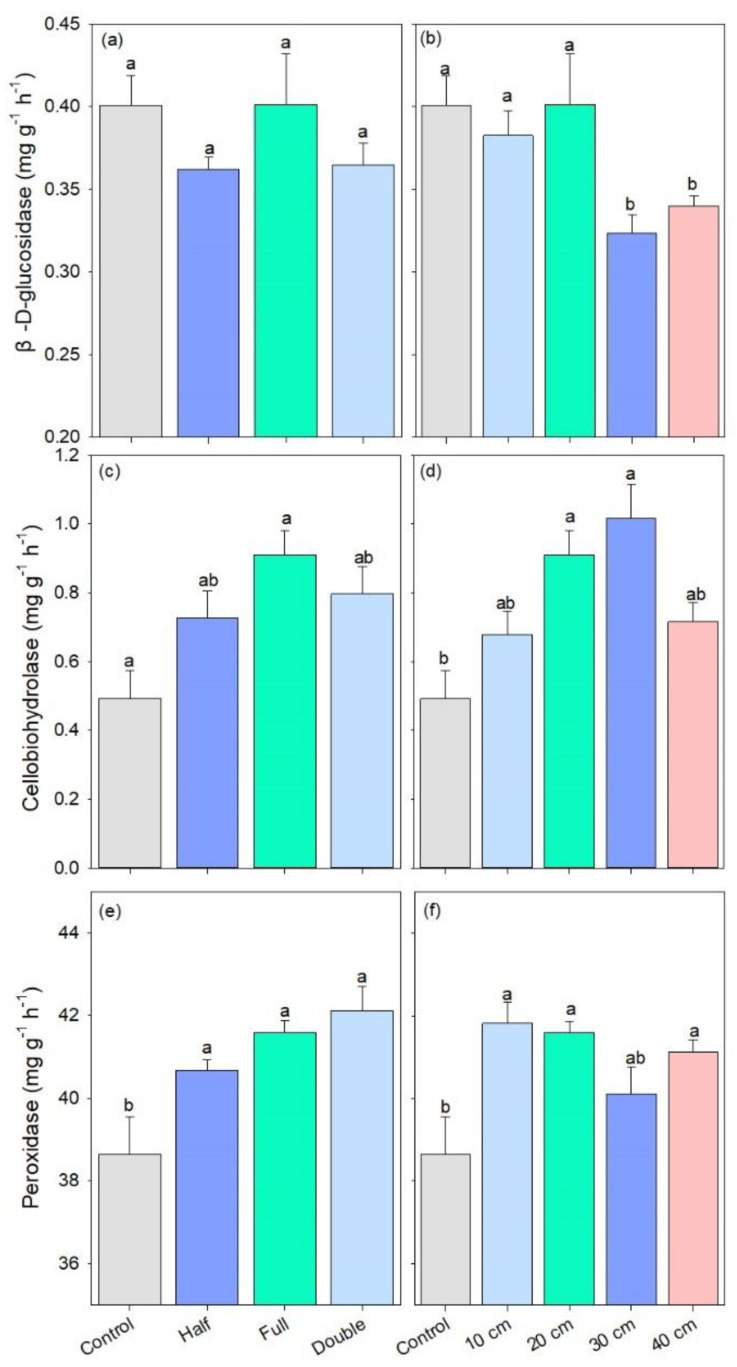
C-degrading enzymatic activity under different straw management practices: (**a**,**b**) β-D-glucosidase; (**c**,**d**) cellobiohydrolase; (**e**,**f**) peroxidase. Control, no tillage and no straw return; 10 cm, straw return with full amount return at a depth of 10 cm; 20 cm, straw return with full amount return at a depth of 20 cm; 30 cm, straw return with full amount return at a depth of 30 cm; 40 cm, straw return with full amount return at a depth of 40 cm; Half, straw return with half amount; Full, straw return with full amount; Double, straw return with double amount. Different letters indicate significant differences between treatments (*p* < 0.05).

The activity of β-D-glucosidase remained stable among straw return amount treatments. However, it was lower under straw buried at 30 and 40 cm as compared to other treatments (*p* < 0.05, Figure 5a,b). DB-SR with full amount increased the activity of cellobiohydrolase by 32% compared to control, and cellobiohydrolase activity was also 28–54% higher under DB-SR at 20 and 30 cm relative to control (*p* < 0.01, Figure 5c,d). In addition, the activity of peroxidase was higher under DB-SR treatments than control, regardless of straw amount or burial depth (*p* < 0.05, Figure 5e,f).

## 4. Discussions

Fungi can decompose organic materials or induce plant disease; as a consequence, the shift in fungal community structure (Figure 1) may influence soil health and crop productivity [15]. Our findings suggested that ditch-buried straw return did not affect fungal diversity (Figure S1), but different burial amounts or depths under ditch-buried straw return structured distinct fungal communities. In particular, saprotrophic and plant pathogenic fungi showed strongly diverse responses to different ditch-buried straw return strategies.

### 4.1. Influences of Ditch-Buried Straw Amount and Depth on Saprotrophic Fungi

Our results showed that DB-SR with full amount increased the abundance of *Ascomycota* as compared to other straw amount treatments (Figure 2a), which has been demonstrated to be capable of degrading cellulose and lignocellulose [34]. Indeed, *Ascomycota* is the prominent saprotrophic fungi in agroecosystems [35]. In soils with higher C contents, DB-SR with a full amount of straw return created a niche that allowed *Ascomycota* to better utilize easily degradable substrates [36]. This explanation is in accordance with Wang et al. [37], who suggested that dense populations of *Ascomycota* mainly affected crop residue decomposition. Similarly, we further observed higher abundances of class *Dothideomycetes* and *Sordariomycetes* under DB-SR with full relative to half and double amounts (Figure S2). These taxa can produce cellulolytic enzymes [38,39]. As a consequence, cellobiohydrolase activity was higher under DB-SR with full amount return relative to half and full (Figure 5c). This is further supported by the positive correlation between the abundance of *Ascomycota* and cellobiohydrolase activity (Figure S3a). These patterns may result from the C demand for fungal growth after straw incorporation.

In agroecosystems, saprotrophic fungi are usually C-limited as abundant N from fertilizers is available [40]. Straw incorporation may reduce C-limitation and therefore induce saprotrophic fungi to secrete more C-acquiring enzymes to degrade such organic residues for their growth [41]. It has been documented that *Sordariomycetes* are involved in straw decomposition and facilitate the transformation of straw to soil organic C by secreting cellulase [42]. From this aspect, DB-SR with the full amount of straw return would increase the exudation of microbial byproducts and subsequently the accumulation of microbial necromass, which facilitates the formation of stable soil C pool [36]. During the process of straw decomposition, nutrients would be released from straw residues. This can be supported by the increased NO_3_^-^ and TN contents under DB-SR with full amounts, as shown in our previous results performed in a similar field [28]. Similarly, our RDA results showed that *Ascomycota* is positively correlated with NO_3_^−^ (Figure 4).

However, with deeper straw burial, the abundance of *Ascomycota* and the activity of β-D-glucopyranoside were lower (Figure 2 and Figure 5), which was contrary to our second hypothesis. This indicates that deeper straw burial may inhibit soil fungal growth and subsequent enzyme production and consequently decrease straw decomposition. Although the growth of soil fungi and the production of enzymes were inhibited when straw returned to deeper soils under DB-SR tillage, it increased the abundance of *Eurotiales* (Figure 2), which might stimulate N_2_O emissions, since the order *Eurotiales* has been demonstrated to be capable of denitrification and therefore potentially producing N_2_O [43]. In short, a larger amount of straw return and deeper buried soil depth would hamper the decomposition of straw as well as the release of energy and nutrients to soils.

### 4.2. Influences of Ditch-Buried Straw Amount and Depth on Plant Pathogenic Fungi

Straw return could provide suitable environments for fungal pathogen growth, reproduction, and accumulation and thus may lead to crop diseases [44]. Here, we found higher relative abundance of *Alternaria* and the predicted plant fungal pathogens under DB-SR with a larger amount and deeper burial (Figure 2c). The genus *Alternaria* consists of plant pathogenic species, which may result in the extensive spoilage of crops [45]. Thus, DB-SR with a larger straw amount or at deeper burial depth may have higher crop disease potential. This could be explained by the fact that a high rate of straw return could influence soil hydrothermal processes [46], which might be favorable to the growth of pathogenic fungi, such as *Alternaria* in soils [45]. On the other hand, many allelochemicals can be released from straw decomposition, such as phenolic acids and short- and long-chain fatty acids, which could decrease specific soil-borne fungal pathogens under a full amount of straw return [47]. Similarly, Zhen et al. [44] reported that the indexes of wheat soil-borne diseases were increased remarkably when the amendment rate increased to 15,000 kg ha^−1^. This was further supported by the higher abundance of dominant *Zygomycota* species like *Mortierella* under full compared to that under the double amount of straw return, since *Mortierella* performs a role in the suppression of disease [48,49].

Moreover, *Fusarium* is the fungal pathogen most responsible for inducing major damage to the production of wheat and maize [50]. Since straw buried below 20 cm increased the abundance of *Fusarium* relative to other buried depth treatments, it may increase the risk of plant pathogens. The results of fungal function prediction determined by the *Fungild* approach also suggested that the predicted plant pathogens were larger whilst arbuscular mycorrhizal fungi were lower under the double amount of straw return and deeper straw (Figure S4). These findings indicate that straw return with the full amount and buried depth less than 30 cm could be beneficial to soil health and crop growth.

Although our case studies showed that ditch-buried straw return with the full amount and buried at a depth less than 30 cm may improve soil nutrient cycles and health and could be beneficial to crop production, higher machinery costs from ditch-buried straw return may exceed the yield benefits. Therefore, it might be beneficial to further explore the economic aspects of implementing deep tillage and straw return, considering the potential costs and farmers’ acceptance.

## 5. Conclusions

Straw return amount and depth under ditch-buried managements influenced the fungal groups with different ecological functions. As compared to half and double burial amounts, DB-SR with a full amount increased the abundance of *Ascomycota* and cellobiohydrolase activity. This in turn, enhanced straw decomposition and subsequently caused higher N availability in soils. Furthermore, the abundance of *Ascomycota* and β-D-glucopyranoside activity were lower with deeper burial depth. Finally, plant pathogenic fungi (i.e., genus *Alternaria* and *Fusarium*) increased with straw burial amount and depth, which may have a negative effect on other fungal community members. Thus, ditch-buried straw return with the full amount and buried at a depth less than 30 cm may improve soil nutrient cycles and health and could be beneficial to crop production.

## Data Availability

The data presented in this study are available on request from the corresponding author.

## Supplementary Materials

The following supporting information can be downloaded at: https://www.mdpi.com/article/10.3390/plants13131738/s1, Figure S1. Shannon diversity (a,b) and chao 1 (c,d) of soil fungi at the phylum and genus level under different straw management practices. Control, no tillage and no straw return; 10 cm, straw return with full amount return at a depth of 10 cm; 20 cm, straw return with full amount return at a depth of 20 cm; 30 cm, straw return with full amount return at a depth of 30 cm; 40 cm, straw return with full amount return at a depth of 40 cm; Half, straw return with half amount; Full, straw return with full amount; Double, straw return with double amount. Different letters indicate significant differences between treatments (*p* < 0.05). Figure S2. Relative abundance of Class Sordariomycetes (a,b) and Dothideomycetes (c,d) under straw management practices. Control, no tillage and no straw return; 10 cm, straw return with full amount return at a depth of 10 cm; 20 cm, straw return with full amount return at a depth of 20 cm; 30 cm, straw return with full amount return at a depth of 30 cm; 40 cm, straw return with full amount return at a depth of 40 cm; Half, straw return with half amount; Full, straw return with full amount; Double, straw return with double amount. Figure S3. Relative abundance of soil fungal functional groups under different straw management practices at guild level according to FUNGuild: (a,c) Plant pathogen; (b,d) Arbuscular mycorrhizal fungi. Control, no tillage and no straw return; 10 cm, straw return with full amount return at a depth of 10 cm; 20 cm, straw return with full amount return at a depth of 20 cm; 30 cm, straw return with full amount return at a depth of 30 cm; 40 cm, straw return with full amount return at a depth of 40 cm; Half, straw return with half amount; Full, straw return with full amount; Double, straw return with double amount. Different letters indicate significant differences between treatments (*p* < 0.05). Table S1 The relative abundance of soil fungal community composition at phylum level under different straw management practices. Control, no tillage and no straw return; 10 cm, straw return with full amount return at a depth of 10 cm; 20 cm, straw return with full amount return at a depth of 20 cm; 30 cm, straw return with full amount return at a depth of 30 cm; 40 cm, straw return with full amount return at a depth of 40 cm; Half, straw return with half amount; Full, straw return with full amount; Double, straw return with double amount. Different letters indicate significant differences between treatments (*p* < 0.05). Figure S4. Correlation between relative abundance of Ascomycete and cellobiohydrolase; and peroxidase across straw management practices.
