# Supplementary material for: Fungal Saprotrophic Promotion and Plant Pathogenic Suppression under Ditch-Buried Straw Return with Appropriate Burial Amount and Depth
Source: Plants (Basel). 2024 Jun 24;13(13):1738. doi: 10.3390/plants13131738 (PMC11243377; doi:10.3390/plants13131738)
Supplement: Supplementary file 1 [file plants-13-01738-s001.zip › plants-2980021-supplementary.pdf]

Supplementary

Materials and methods

DNA extraction

The DNA was extracted from 0.5 g of each soil sample using a Fast DNA SPIN Kit for Soil (Illumina, San Diego, CA, USA). DNA quality was examined by 1.5% agarose gel electrophoresis in 1 × TAE buffer, and the DNA concentration was quantified using an ND-1000 spectrophotometer (Nanodrop Technology, Wilmington, USA). The resultant DNA samples were stored at -80 °C prior to PCR amplification. The fungal ITS rDNA gene was amplified with the primer pair ITS1F (5′-barcode-CTTGGTCATTTAGAGG AAGTAA-3′) and 2043R (5′-GCTGCGTTCT TCATCGATGC-3′) (White et al., 1990). The barcode was an eight-base sequence unique to each sample. The PCR was implemented in 20 μl reaction mix containing 4 μl 5 × Fast Pfu buffer, 2 μl dNTPs (2.5 mM), 0.8 μl forward primer (5 μM) and reverse primer (5 μM), 0.4 ml Fast Pfu polymerase, 0.2 μl BSA, and 10 ng template DNA. The PCR reaction procedure was as follows: initial incubation at 95 °C for 3 min, 33 cycles of 95 °C for 30s, 55 °C for 30s, and 72 °C for 45 s, with a final extension step at 72 °C for 10 min. The PCR amplicons were purified with the QIAquick PCR purification kit (QIANGEN). Amplicons were extracted from 2% agarose gel and purified for twice using the AxyPrep DNA Gel Extraction Kit (Axygen Biosciences, Union City, CA, U.S.) according to the manufacturer's instructions and quantified using QuantiFluor™-ST (Promega, U.S.). Purified amplicons were pooled in equimolar amounts and paired-end sequenced (2 × 250) on an Illumina MiSeq platform according to the standard protocols. Operational taxonomic units (OTUs) were clustered with 97% similarity cutoff using

UPARSE (version 7.1 http://drive5.com/uparse/) and chimeric sequences were identified and removed using UCHIME (Edgar et al., 2011).

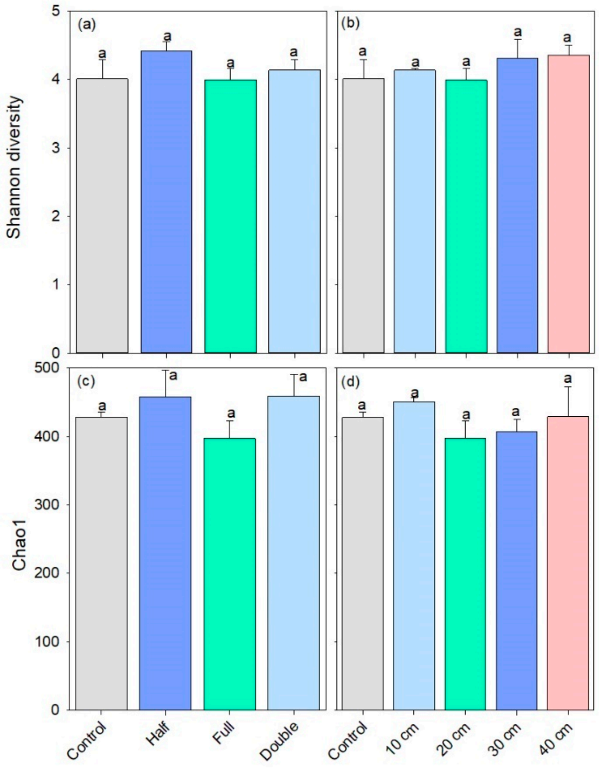

Fig. S1 Shannon diversity (a, b) and chao 1 (c, d) of soil fungi at the phylum and genus level under different straw management practices. Control, no tillage and no straw return; 10 cm, straw return with full amount return at a depth of 10 cm; 20 cm, straw return with full amount return at a depth of 20 cm; 30 cm, straw return with full amount return at a depth of 30 cm; 40 cm, straw return with full amount return at a depth of 40 cm; Half, straw return with half amount; Full, straw return with full amount; Double, straw return with double amount. Different letters indicate significant differences between treatments ($p < 0.05$).

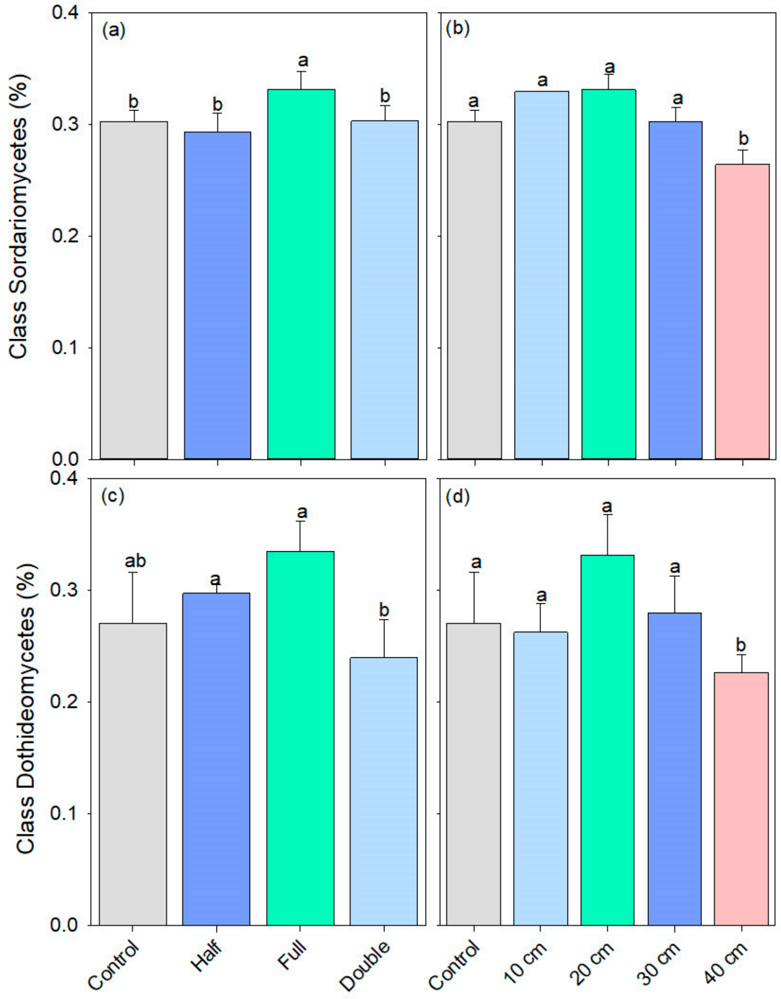

Fig. S2 Relative abundance of Class Sordariomycetes (a, b) and Dothideomycetes (c, d) under straw management practices. Control, no tillage and no straw return; 10 cm, straw return with full amount return at a depth of 10 cm; 20 cm, straw return with full amount return at a depth of 20 cm; 30 cm, straw return with full amount return at a depth of 30 cm; 40 cm, straw return with full amount return at a depth of 40 cm; Half, straw return with half amount; Full, straw return with full amount; Double, straw return with double amount.

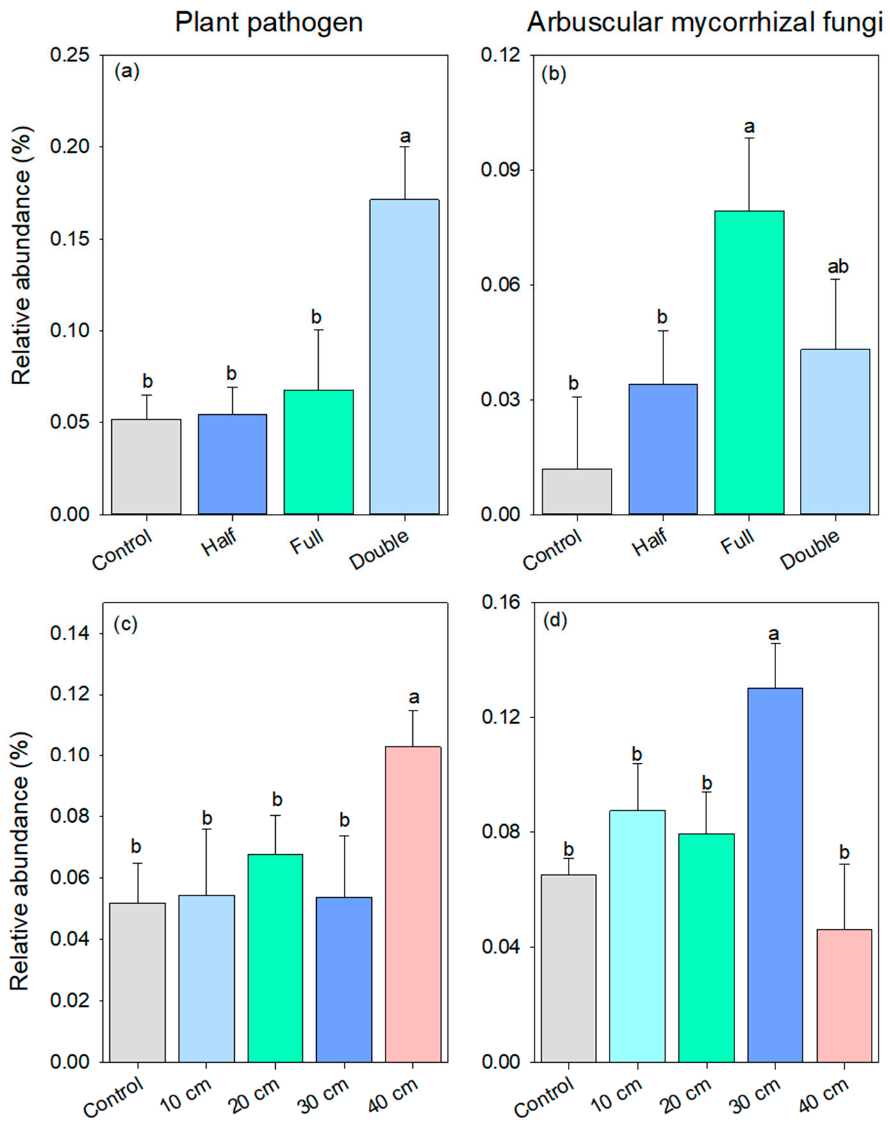

Figure S3. Relative abundance of soil fungal functional groups under different straw management practices at guild level according to FUNGuild: (a, c) Plant pathogen; (b, d) Arbuscular mycorrhizal fungi. Control, no tillage and no straw return; 10 cm, straw return with full amount return at a depth of 10 cm; 20 cm, straw return with full amount return at a depth of 20 cm; 30 cm, straw return with full amount return at a depth of 30 cm; 40 cm, straw return with full amount return at a depth of 40 cm; Half, straw return with half amount; Full, straw return with full amount; Double, straw return with double amount. Different letters indicate significant differences between treatments ($p < 0.05$).

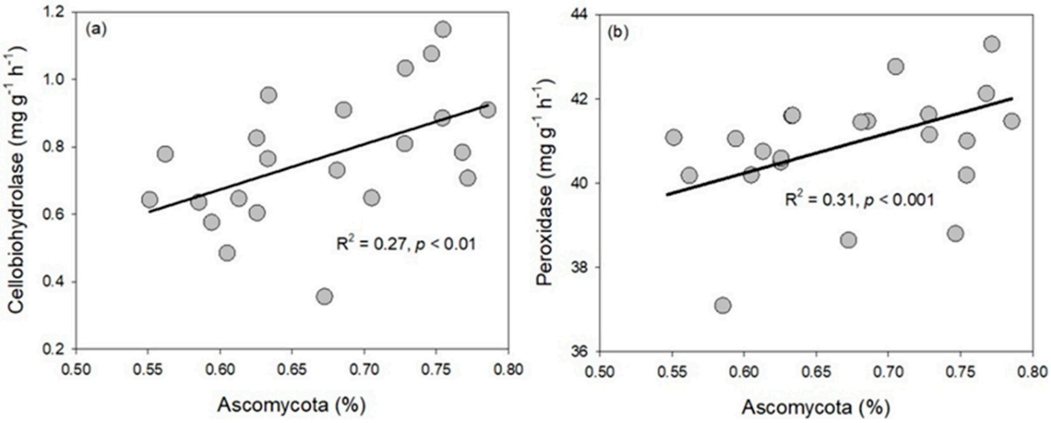

Figure S4. Correlation between relative abundance of Ascomycete and cellobiohydrolase; and peroxidase across straw management practices.

Table S1 The relative abundance of soil fungal community composition at phylum level under different straw management practices. Control, no tillage and no straw return; 10 cm, straw return with full amount return at a depth of 10 cm; 20 cm, straw return with full amount return at a depth of 20 cm; 30 cm, straw return with full amount return at a depth of 30 cm; 40 cm, straw return with full amount return at a depth of 40 cm; Half, straw return with half amount; Full, straw return with full amount; Double, straw return with double amount. Different letters indicate significant differences between treatments ($p < 0.05$).

	Ascomycota	Basidiomycota	Chytridiomycota	Glomeromycota	Zygomycota
Control	67±2.3b	5.9±0.6	0.0±0.0a	0.0±0.0b	1.4±0.0b
Half	69±4.1ab	11±2.7a	0.0±0.0a	14.7±1.1a	0.0±0.0c
Full	73±2.1a	9.6±1.5b	0.0±0.0a	0.0±0.0b	2.1±0.0a
Double	68±6ab	7.5±1.5bc	0.1±0.0a	0.06±0.0b	1.7±0.1b
Control	67±2.0b	11±2.7a	0.0±0.0a	0.0±0.0a	1.4±0.0c
10 cm	61±2.2b	8.2±0.9b	0.0±0.0a	0.0±0.0a	2.1±0.3b
20 cm	73±2.3a	5.9±0.6c	0.0±0.0a	0.0±0.0a	1.5±0.3c
30 cm	64±6b	7.7±1.3bc	0.0±0.0a	0.0±0.0a	2.1±0.1b
40 cm	63±6.0b	5.9±0.6c	0.0±0.0a	0.0±0.0a	3.8±0.8a
